# Optogenetic Control of Heart Rhythm by Selective Stimulation of Cardiomyocytes Derived from Pnmt^+^ Cells in Murine Heart

**DOI:** 10.1038/srep40687

**Published:** 2017-01-13

**Authors:** Yanwen Wang, Wee Khang Lin, William Crawford, Haibo Ni, Emma L. Bolton, Huma Khan, Julia Shanks, Gil Bub, Xin Wang, David J. Paterson, Henggui Zhang, Antony Galione, Steven N. Ebert, Derek A. Terrar, Ming Lei

**Affiliations:** 1 Department of Pharmacology, University of Oxford, Oxford, UK; 2 University of Queensland, Brisbane, Australia; 3 School of Physics and Astronomy, University of Manchester, Manchester, UK; 4 Department of Physiology, Anatomy and Genetics, University of Oxford, Oxford, UK; 5 Faculty of Life Science, University of Manchester, Manchester, UK; 6 Burnett School of Biomedical Sciences, College of Medicine, University of Central Florida, Orlando, USA

## Abstract

In the present study, channelrhodopsin 2 (ChR2) was specifically introduced into murine cells expressing the *Phenylethanolamine n-methyltransferase (Pnmt*) gene, which encodes for the enzyme responsible for conversion of noradrenaline to adrenaline. The new murine model enabled the identification of a distinctive class of Pnmt-expressing neuroendocrine cells and their descendants (i.e. Pnmt^+^ cell derived cells) within the heart. Here, we show that Pnmt^+^ cells predominantly localized to the left side of the adult heart. Remarkably, many of the Pnmt^+^ cells in the left atrium and ventricle appeared to be working cardiomyocytes based on their morphological appearance and functional properties. These Pnmt^+^ cell derived cardiomyocytes (PdCMs) are similar to conventional myocytes in morphological, electrical and contractile properties. By stimulating PdCMs selectively with blue light, we were able to control cardiac rhythm in the whole heart, isolated tissue preparations and single cardiomyocytes. Our new murine model effectively demonstrates functional dissection of cardiomyocyte subpopulations using optogenetics, and opens new frontiers of exploration into their physiological roles in normal heart function as well as their potential application for selective cardiac repair and regeneration strategies.

A major challenge faced in biomedical research is to define the function of specific types of cells in a highly complex multicellular system such as the brain or heart. This requires the ability to control the activity of one type of cell with cellular precision while leaving all other cells unaltered. Adrenergic cells produce the neurotransmitter/hormone adrenaline in the brain (e.g. ventrolateral medulla) and peripheral tissues (e.g. adrenal medulla). These cells play a variety of roles, particularly in stress responses such as ‘fight or flight’. A key feature and marker of these cells is the expression of phenylethanolamine N-methyltransferase (Pnmt), the enzyme responsible for conversion of noradrenaline to adrenaline^1^. Intriguingly, Pnmt was also found to be expressed in the heart from the early embryonic stage to adulthood. During early embryonic development, Pnmt expression first appears in association with the pacemaking and conduction system^1^. There is a transient surge of Pnmt expression when the first myocardial contraction occurs before any nerve-like or neural crest cells appear in the heart^2^. Such early expression of Pnmt is believed to denote a mesodermal origin of adrenergic cells in the heart giving rise to so called “Intrinsic Cardiac Adrenergic” (ICA) cells that were first reported in 1990s^2^,^3^. Somewhat later in development, neural crest-derived (NCD) cells invade the heart and also appear to contribute to neuroendocrine functions^1^. The physiological role of these neuroendocrine cells within the heart, however, is still poorly understood.

To evaluate the developmental localization of adrenergic cells in the heart, we previously developed a mouse line (*Pnmt-Cre* mice) by inserting the *Cre-recombinase* gene into the mouse *Pnmt* locus so that the expression of Cre-recombinase depends upon *Pnmt* regulatory sequences. The mice were then cross-mated with homozygous Rosa26 reporter (R26R) mice^4^. Because Pnmt is a marker for adrenergic cells, expression of β-galactosidase (βGAL) marked cells of an adrenergic lineage in the offspring. Using this strategy, we traced Pnmt^+^ cells and their descendants in murine developing and adult hearts^2^, and found that the distribution of these Pnmt^+^ cells and their descendants shows a wide spread in all four cardiac chambers through fetal and neonatal development periods, but remarkably becomes largely restricted to the left side of the heart by adult stages^5^. βGAL expression was found in a substantial fraction of cardiomyocyte-like cells, suggesting that the possibility of significant numbers of cardiomyocytes were derived from an adrenergic lineage^5^.

Recently there has been a resurgence of interest in the intrinsic cardiac adrenergic system, as several studies have demonstrated the potential importance of these cells for cardiac regeneration and sympathetic re-innervation following cardiac transplantation^6^,^7^,^8^. Consequently, it is important to develop a better understanding of the physiological roles of these cells *in vivo*.

In the present study, we address this issue by adopting an optogenetic approach to study the electrophysiological properties of Pnmt-derived cardiomyocytes (PdCMs). Emerging optogenetic techniques provide an unprecedented opportunity to address such a challenge by delivering optogenetic control with spatiotemporal resolution and cell type–specific manner precision. Since the first demonstrations of the utility of this approach in mammalian neurons in 2005^9^,^10^, optogenetic methods have spurred immense research activity in neuroscience and have been recently been extended into the cardiac field^11^,^12^,^13^,^14^,^15^,^16^,^17^,^18^. In the present study, we generated a new mouse strain by crossing Pnmt-Cre mice with Ai27D mice (Stock No. 012567, Jackson Labs)^19^ expressing an improved channelrhodopsin-2/tdTomato fusion protein. Following exposure to Cre recombinase in PdCMs, the offspring mice were used in optogenetic studies for rapid activation of excitable cells by illumination with blue light (450–490 nm). This allowed us to study their physiological properties *in* intact cardiac tissue for the first time.

We used the resulting mice to study the electrophysiological properties of PdCMs. Our study demonstrates a significant expression of PdCMs in specific regions of the heart where they respond to optogenetic stimulation. Therefore, the new Pnmt-Cre/ChR2 mice provide a valuable tool for understanding physiological properties of PdCMs by selectively stimulating them in the intact heart with high spatiotemporal resolution. Thus our study points a new direction for functional dissection of cardiomyocyte subpopulations using optogenetics in the heart.

## Results

### Generation of optogenetic cell type specific murine model and immuno-histological characterizations

**Figure 1:**
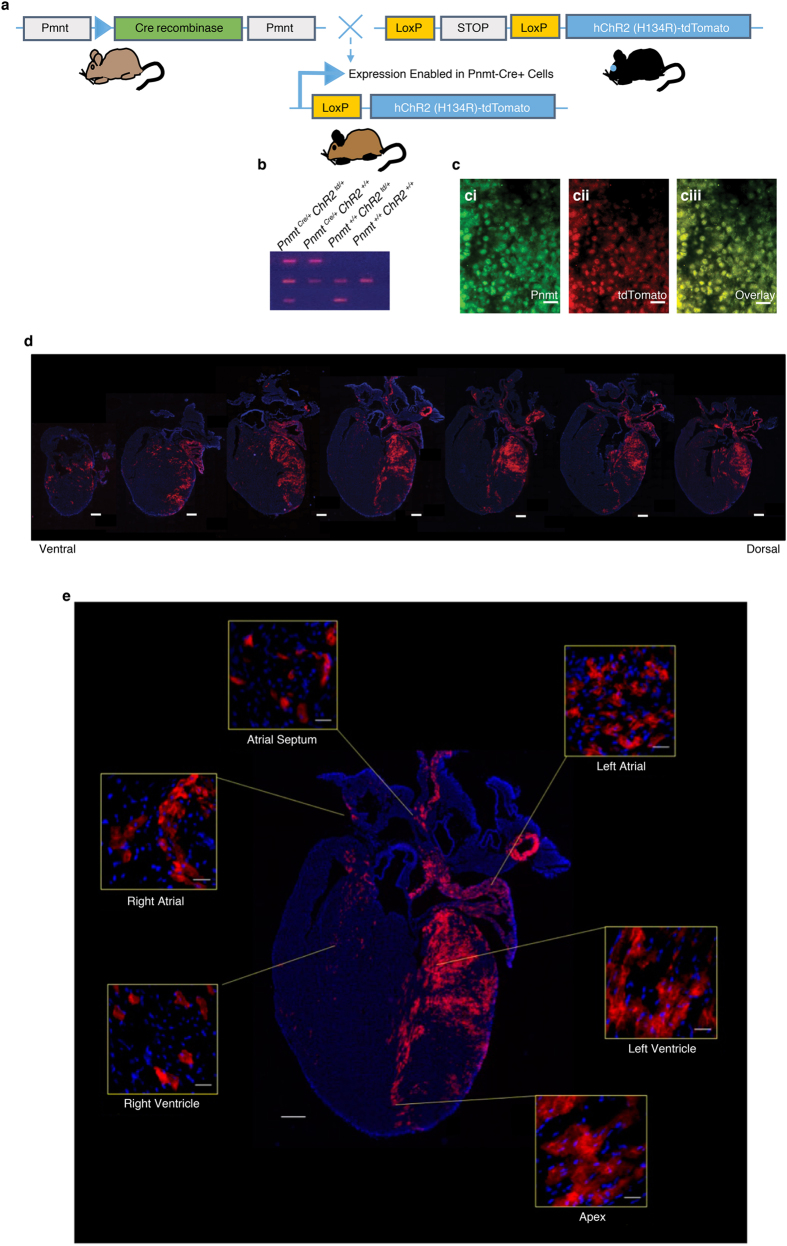
Development of a mouse model of adrenergic cell-type specific expression of ChR2 and histological characterisation. (**a**) Schematic diagram demonstrating cell-type specific expression of ChR2-tdTomato under a *Pnmt* promoter. (**b**) Genotypes of offspring showing successful cell-type specific expression of ChR2-tdTomato in *Pnmt*^*Cre/ChR2*^ animals. (**c**) Immunostaining of *Pnmt* with anti-Pnmt antibody (ci); tdTomato fluorescence in a representative section of *Pnmt*^*Cre/ChR2*^ adrenal medulla (cii); overlay of ci and cii, showing the co-localisation of Pnmt and tdTomato fluorescence (n = 5 hearts). (**d**) Tdtomato fluorescence in multiple coronal sections of adult *Pnmt*^*Cre/ChR2*^ mouse heart demonstrating the distribution of PdCMs throughout the whole heart (n = 10 hearts). (**e**) A representative image showing TdTomato fluorescence of the coronal section taken from an adult *Pnmt*^*Cre/ChR2*^ mouse heart, with inserts showing the tdTomato fluorescence in different regions of the heart. Scale bars: c: 50 μm; d and e: 500 μm; inserts in e: 100 μm.

A new mouse line was generated by crossing Pnmt-Cre mice^4^ with B6.Cg-*Gt (ROSA)26Sor*^*tm27.1(CAG-COP4H134R/tdTomato) Hze*^/J strain (Stock No. 012567, Jackson Labs) (Fig. 1a). The new mouse line provides an optogenetic tool for studying Pnmt^+^ cells and their descendants. Genotyping was conducted to differentiate mice with the genotype of *Pnmt*^*Cre/ChR2*^ with both Pnmt-Cre and ChR2/tdTomato expression and *Pnmt*^+/+^ mice that did not express Pnmt-Cre but did contain the latent ChR2/tdTomato gene (Fig. 1b). Pnmt immunostaining and tdTomato fluorescence were examined in adrenal medulla sections of *Pnmt*^*Cre/ChR2*^ mice to confirm co-expression of Pnmt and tdTomato (Fig. 1c). The tdTomato and ChR2 are a fusion protein in Ai27D line^19^, and the distribution of cells expressing ChR2/tdTomato was therefore examined in adult *Pnmt*^*Cre/ChR2*^ hearts by detecting tdTomato or ChR2 fluorescence. TdTomato fluorescence or ChR2 staining in adult *Pnmt*^*Cre/ChR2*^ heart coronal sections revealed a distinct localized expression of ChR2/tdTomato-positive cells (Fig. 1d and e). Particularly high expression of ChR2/tdTomato-positive cells was noted in the left atrium (LA), left ventricle (LV) and intraventricular septum (Fig. 1e). Among ChR2/tdTomato-positive cells, 86 ± 8% were found on the left side of the heart (p = 0.02; n = 4 hearts analysed, unpaired *t* test).

**Figure 2:**
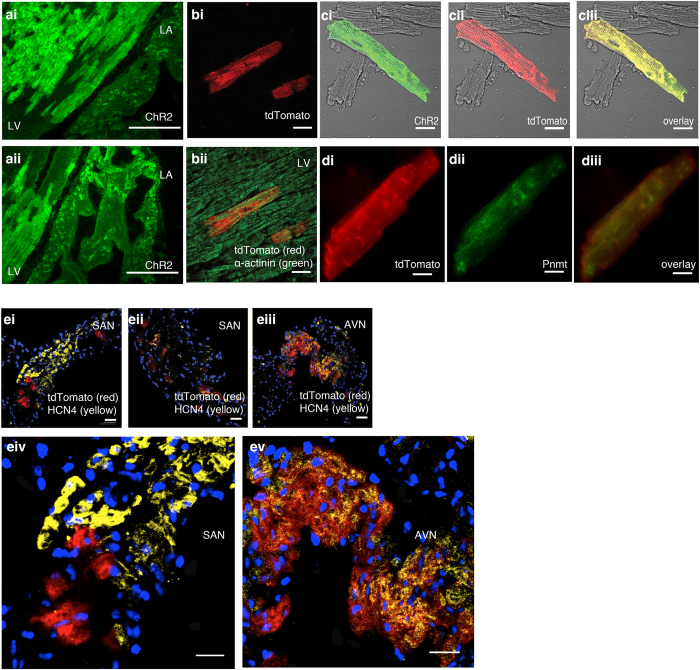
Immunohistological and immunocytochemistrical characterizations. (**a**) ChR2 staining in LV and LA sections from adult *Pnmt*^*Cre/ChR2*^ mouse heart (n = 4 hearts). The majority of Pnmt-derived cells are located on the left ventricle and left atrium. (**b**) TdTomato fluorescence without (bi) and with (bii) anti-α-actinin antibody staining in the left ventricle of a coronal heart section. (**c**) Immunostaining of ChR2 with anti-ChR2 antibody (ci); tdTomato fluorescence in isolated LV cardiomyocytes (cii); overlay of ci and cii, showing co-localisation of ChR2 and tdTomato (n = 8 cells) (ciii). (**d**) Immunostaining of ChR2 with anti-ChR2 antibody (di); Pnmt staining in isolated LV cardiomyocytes (dii); overlay of di and dii, showing co-expression of ChR2 and Pnmt (diii) (n = 4 cells). (**e**) Transverse sections from SAN (ei, eii) and AVN (eiii) regions stained with anti-HCN4 antibody. eiv and ev show the enlarged imaged of ei and eiii respectively. Co-localisation of HCN4-tdTomato fluorescence is shown in orange. (n = 4 preprations). Scale bars: a: 100 μm; b: 20 μm; c: 30 μm; d: 20 μm; e: 30 μm (i, ii, iii, iv and v).

Further investigation demonstrated the expression of ChR2/tdTomato fusion protein by detecting the co-localization of ChR2 immunoreactivity and tdTomato fluorescence in specific myocytes (ChR2/tdTomato-positive cells) but not conventional myocytes (Fig. 2a–c), indicating the suitability of tdTomato as a convenient fluorescence marker for cells expressing ChR2.

A large number of cardiomyocyte-like cells were found to be localized in the left side of the heart and conduction system (Fig. 2a and e). Estimated percentages of ChR2/tdTomato-positive cardiac myocytes in LA and LV are account approximately 50% and 21% of the total myocytes in these regions, while the estimated percentages of ChR2/tdTomato-positive cardiac myocytes in RA (free wall) and RV are only approximately 7% and 2% of the total myocytes in these regions. A large number of ChR2/tdTomato-positive cells were also localized to areas of sinoatrial node (SAN) and atrioventricular node (AVN) (Fig. 2e). Immunocytochemistry studies were carried out to show the colocalisation of CHR2 staining/TdTomato fluorescence and Pnmt staining (Fig. 2d). Cardiomyocyte identity was further confirmed by morphology, positive expression of α-actinin (a marker of contractile myocytes) (Fig. 2b,c), and expression of HCN4 (pacemaker cell marker) (Fig. 2e). There was a large number of cells with co-expression of tdTomato and HCN4 in the AVN region but few in the SAN region.

A three-dimensional model to represent ChR2/tdTomato fluorescence is shown in online video 1 demonstrating the cell distribution throughout the ventricle. Interestingly, Pmnt staining was observed in some PdCMs (~10% of isolated ventricular PdCMs) (Fig. 2d).

### Optogenetic electrophysiological characterization

**Figure 3:**
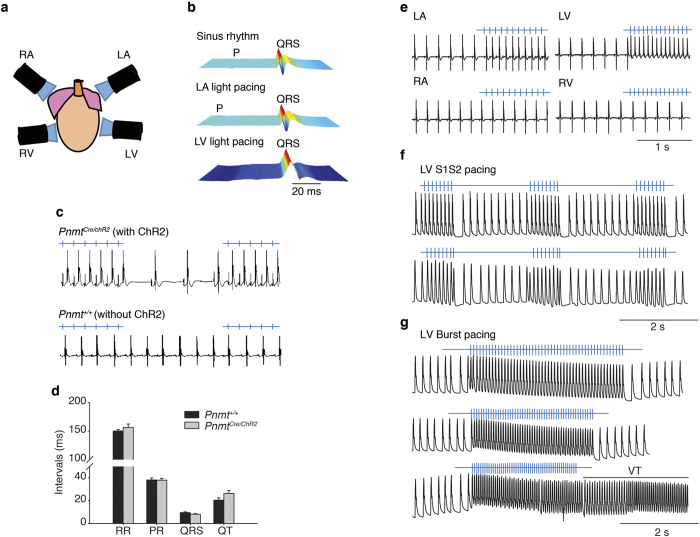
Controlling heart rhythm with selective optogenetic stimulation of PdCMs. (**a**) Localized light pacing in 4 different regions of the heart with blue light. (**b**) Upper panel shows a representative ECG recording of a *Pnmt*^*Cre/ChR2*^ heart in intrinsic sinus rhythm. The middle and bottom panels show representative ECG recordings of this heart paced by targeting blue light pulses to the LA (middle) and LV (lower) regions respectively. “P” in representative ECG recordings stands “P wave”. (**c**) Light pulses delivered to the LA of a *Pnmt*^*Cre/ChR2*^ heart evoked ECG spikes in a 1:1 manner. In contrast, wild-type *Pnmt*^+/+^ hearts show no response to light pulses (n = 8 hearts). (**d**) Comparison of multiple ECG parameters, including RR interval, PR interval, QRS interval and QT interval. No significant difference was noted between *Pnmt*^*Cre/ChR2*^ and *Pnmt*^+/+^ hearts for any ECG parameter (n = 8 hearts, *P* > 0.05). (**e**) Representative ECG recordings from LA, LV, RA, RV showing effects of local light stimulation (2–10 ms pulses, frequency 8–10 Hz) with the light beam placed close to the epicardium in different regions of a *Pnmt*^*Cre/ChR2*^ heart (n = 8 hearts). (**f**,**g**) MAPs recorded in LV of a *Pnmt*^*Cre/ChR2*^ heart by programmed light stimulation (PLS) with S1S2 protocol (**f**) or burst pacing protocol (**g**). Light pulses were delivered to the LV region in this experiment (n = 8 hearts). Light intensity for these experiments is 0.6–0.8 mW/mm^2^.

We then determined the functional role of PdCMs using light pulses to selectively stimulate these cells by activation of ChR2 channels in adult *Pnmt*^*Cre/ChR2*^ hearts. We determined whether the intrinsic rhythm of *Pnmt*^*Cre/ChR2*^ hearts could be overridden by stimulating PdCMs using light pacing. Spatiotemporally controlled stimulation of PdCMs in intact heart was achieved using light pulses (470 nm wavelength, 2 ms duration) generated by a Transistor-transistor logic (TTL) -controlled light emitting diode (LED) directed towards Langendorff-perfused *ex vivo* hearts (Fig. 3a). Ventricular ECGs and/or monophasic action potentials (MAP) were recorded simultaneously. Online video 2 shows light pacing overriding the intrinsic sinus rhythm in a *Pnmt*^*Cre/ChR2*^ heart. ECGs indicative of atrial and ventricular pacing were recorded in *Pnmt*^*Cre/ChR2*^ hearts when light pulses were delivered to the LA and LV (Fig. 3b). Light pulses delivered to the LA of *Pnmt*^*Cre/ChR2*^ hearts, but not *Pnmt*^+/+^ hearts evoked ECG spikes in a 1:1 manner (Fig. 3c, n = 8 hearts). There were no significant differences in ECG characteristics between *Pnmt*^+/+^ hearts (RR interval: 150.0 ± 7.0 ms, PR interval: 38.6 ± 5.0 ms; QRS interval: 9.5 ± 1.8 ms; QT interval: 20.5 ± 5.9 ms, n = 8 hearts) and *Pnmt*^*Cre/ChR2*^ hearts (RR interval: 156.4 ± 17 ms, PR interval: 39.0 ± 5.6 ms; QRS interval: 8.1 ± 1.6 ms; QT interval: 24 ± 7.0 ms, n = 8 hearts, Mean ± SD, *Pnmt*^+/+^ vs *Pnmt*^*Cre/ChR2*^ hearts, p > 0.05, unpaired *t* test) at intrinsic sinus rhythm (Fig. 3d). We next evaluated the capture of cardiac rhythm by pacing different regions of the *Pnmt*^*Cre/ChR2*^ heart. Figure 3e shows the recordings of ECGs in *Pnmt*^*Cre/ChR2*^ hearts under conditions of intrinsic sinus rhythm, or light pacing targeted to different regions of the heart. Remarkably, the capture of sinus rhythm was highly correlated to the distribution pattern of PdCMs. In *Pnmt*^*Cre/ChR2*^ hearts, pulsed light targeted to the LA (Fig. 3e, top left panel), but not RA (bottom left panel), led to an ECG with a P-R interval that was indicative of supraventricular pacing (triggered by light pulses). This is comparable to a P-R interval during intrinsic sinus rhythm. The morphology of the QRS waveform and QT intervals of light-paced hearts on the left atrium and hearts in intrinsic sinus rhythm were comparable. The characteristics of ECG waveforms between sinus rhythm and light pacing induced rhythm (measured at CL 100 ms pacing frequency) in *Pnmt*^*Cre/ChR2*^ hearts were similar. Light pacing: PR interval: 37.1 ± 3.1 ms; QRS interval: 10 ± 4.8 ms, QT interval: 23.8 ± 7.2 ms; Sinus rhythm: PR interval: 39.0 ± 5.6 ms; QRS interval: 8.1 ± 1.6 ms; QT interval: 24 ± 7.0 ms, n = 8 hearts (Mean ± SD, light pacing vs. sinus rhythm, p > 0.05, paired *t* test).

Consistently, pulsed illumination of LV (Fig. 2e upper right panel), but not RV (Fig. 3e bottom right panel) led to ECGs indicative of ventricular pacing. Programmed light stimulation (PLS) protocols also allowed us to examine electrophysiological properties of the in *Pnmt*^*Cre/ChR2*^ hearts (n = 8 hearts) by recording MAPs as shown in Fig. 3f and g. Consistent with the observations of ECG characteristics, the MAP morphology and characteristics were similar between sinus rhythm and light pacing induced rhythm in the same region of the heart. For example, APD_90_ measured un sinus rhythm and at CL 100 ms pacing frequency from LV free wall recording site are: 113 ± 21 ms (sinus rhythm), 102 ± 37.8 ms (light pacing) (Mean ± SD, light pacing vs. sinus rhythm, n = 7, p > 0.05, paird *t* test). Using this approach we were able to effective refractory period of the region stimulated by the light, conduction properties and the arrhythmic susceptibility of the heart under acute β-adrenergic stress provokes by the perfusion of the heart with 50 nM isoprenaline (ISO). The observations were similar to those made under similar conditions using conventional programmed electrical stimulation^20^. In such cases, two light Programmed light stimulation (PLS) protocols were conducted: (i) A pacing train of eight stimuli (S1) was delivered at a basic cycle length of 100 ms, with a single (S2) premature extra stimulus introduced at progressively shorter intervals until arrhythmia was induced or the ventricular effective refractory period (VERP) was reached (as in the examples shown in Fig. 3f) (ii). Burst pacing protocols used either 50 variable frequency S1S1 cycle lengths from 100 ms to 30 ms or 50 fixed cycle lengths (20 ms), as in the examples shown in Fig. 3g. To assess the propensity to ventricular arrhythmias of Langendorff-perfused hearts under baseline conditions or acute ISO (50 nM) treatment, the hearts were subjected to PLS as in the examples shown in Fig. 3g bottom panel. Ventricular tachycardia was defined as the occurrence of six or more consecutive premature ventricular waveforms; tachycardia with regular waveforms was defined as VT, and VF was characterized by irregular fibrillating waveforms. The same S1S2 and S1S1 protocols conducted by programmed electrical stimulation (PES) were also conducted in some of these hearts (5 out 8).

**Figure 4:**
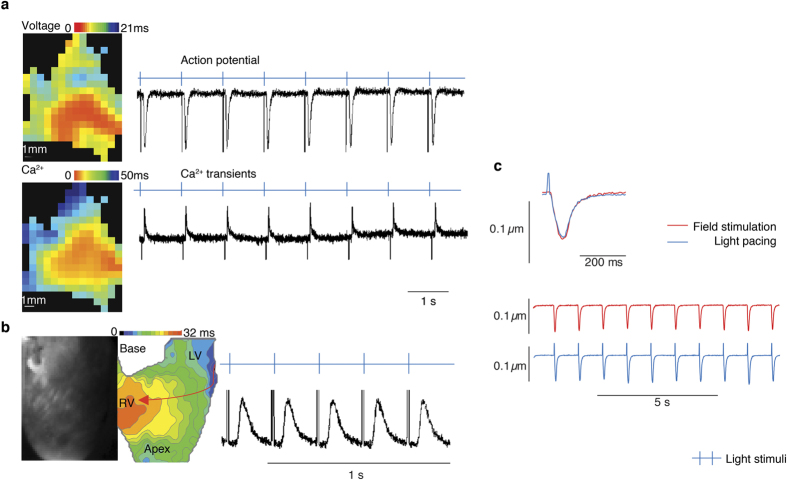
(**a**) Activation maps (left) and traces (right) of Ca^2+^ transients (upper) and action potentials (lower) obtained from a light-paced LA isolated from a *Pnmt*^*Cre/ChR2*^ heart and pre-incubated with Rhod-2 AM (Ca^2+^ dye) and RH237 (voltage dye). Light pulses evoked Ca^2+^ transients and action potentials in a 1:1 manner (n = 6 hearts). Light intensity: 0.6–0.8 mW/mm^2^. (**b**) Representative image (left), light pacing induced global Ca^2+^ transient activation map (middle) and fluorescence traces (right) of optical Ca^2+^ transients obtained from light paced LV slices from a *Pnmt*^*Cre/ChR2*^ heart that were pre-incubated with Rhod 2-AM (Ca^2+^ dye) and RH237 (voltage dye). Light pacing resulted in an anisotropic propagation spread from LV to RV (n = 6 hearts). Light intensity: 0.6–0.8 mW/mm^2^. (**c**) Representative sarcomere shortening (cardiomyocyte contraction) traces triggered by electric pulses (upper) and light pulses (middle) in a single PdCM isolated from a *Pnmt*^*Cre/ChR2*^ heart (n = 6 hearts). The lower panel shows superimposition of sarcomere shortening traces triggered by electric and light pulses.

The electrophysiological and Ca^2+^ handling properties of PdCMs at tissue and single cell levels in *Pnmt*^*Cre/ChR2*^ hearts were examined by combining light pacing with simultaneous monitoring of voltage and Ca^2+^ signals by optical imaging in dissected LA preparations and whole hearts from *Pnmt*^*Cre/ChR2*^ mice. The preparations were loaded with a voltage-sensitive dye RH237 and/or Ca^2+^ indicator Rhod-2 AM. Figure 4a shows the activation maps of action potentials and Ca^2+^ transients evoked from a light-paced LA dissected from a *Pnmt*^*Cre/ChR2*^ heart. Following light pacing, the action potential duration was 37 ± 2 ms at 50% repolarization (APD_50_) and 83 ± 9 ms at 90% repolarization (APD_90_) (n = 6 LA preparations). These values are comparable to action potential durations recorded from mouse hearts during electrical stimulation. Ca^2+^ transients stimulated by light pulses were also obtained in LV (n = 6 hearts). Representative traces and a global Ca^2+^ transient activation map of optically imaged Ca^2+^ transients in light-paced LV are shown in Fig. 4b. In addition, contractions were recorded using light to stimulate single PdCMs isolated from the *Pnmt*^*Cre/ChR2*^ heart as demonstrated in Online video 3. Figure 4c demonstrates mechanical responses triggered by stimulation of ChR2 with light in single cells (n = 6 cells).

## Discussion

Our results demonstrate a compelling case for the use of optogenetic approaches to control heart rhythm by selectively stimulating PdCMs in adult hearts. The combined genetic expression of a fluorescence tag and ChR2 proteins under a cell type-specific promoter provides the advantage of simultaneous cell-type selective functional stimulation and live-cell imaging. We anticipate such cell-type specific optogenetic approaches will be a starting point for developing sophisticated model systems for addressing unmet needs in heart research, in particular, combining with other Cre mice with optogenetic reporter mice, to study the other subpopulations within the heart.

PdCMs are predominantly localized in the SAN, AVN and left side of the heart. In SAN, they present in peripheral rather than central regions, while in AVN, a substantial number were also HCN4 positive. Co-localization of Pnmt (βGAL) and HCN4 were previously demonstrated in embryonic E15.5 mouse heart^4^. The distribution pattern of PdCMs in adult *Pnmt*^*Cre/ChR2*^ mice reported in this study was consistent with our previous study on *βGAL* expression in *Pnmt*^+/*Cre*^ *ROSA26*^+*/βgal*^ mice, in which βGAL expression was predominantly (89%) located in the LA and LV, where many of these *βGAL* positive cells appeared to have cardiomyocyte-like morphological and structural characteristics^5^. The staining pattern in the LA was diffuse, but the LV free wall displayed intermittent non-random staining that extended from the apex to the base of the heart, including heavy staining of the anterior papillary muscle along its perimeter^5^. Thus the *Pnmt*^+*/Cre*^ *ROSA26*^+*/βgal*^ mouse provides a valuable model system for a detailed anatomical characterization of the fate of adrenergic cells in the adult mouse heart through identifying the cells marked by *βGAL* expression in this model. Based on our current results, we suggest that those cardiomyocyte-like *βGAL* positive cells described in our previous study^5^ are PdCMs. It is unclear why PdCMs are predominantly expressed in the left side of the heart and conduction system. One possible explanation is that their distribution reflects their important role in these regions, and this needs to be further explored. Indeed, it was previously shown that adrenergic deficiency led to impaired electrical conduction and increased arrhythmic potential in embryonic mouse heart^21^. Our new Pnmt-Cre/ChR2 mice provide a valuable functional tool for the study of the physiological properties of PdCMs by selectively stimulating them in the intact heart with high spatiotemporal resolution, which can not be achieved by *Pnmt*^+*/Cre*^ *ROSA26*^+*/βgal*^ mice. We then determined the functional role of PdCMs using light pulses to selectively stimulate these cells by activation of ChR2 channels in adult *Pnmt*^*Cre/ChR2*^ hearts and determined whether the intrinsic rhythm of *Pnmt*^*Cre/ChR2*^ hearts could be overridden by stimulating PdCMs using light pacing.

The number of PdCMs in LA is sufficient to trigger a change in rhythm of the atria and ventricles when these PdCM cells are activated by light pulses. Optical imaging of voltage and Ca^2+^ signals evoked by light pulses in LA or LV confirmed the effectiveness of light pacing to generate electrical excitation and propagation in tissue, comparable to activity initiated by conventional electrical stimulation. The mechanical response of single PdCMs triggered by light stimulation illustrates the direct optogenetic control of contraction of specific cardiomyocytes expressing ChR2. Consequently, these results confirm for the first time that some Pnmt-derived cells in the adult mouse heart are, in fact, functional cardiomyocytes. Prior studies have shown that these cells express contractile proteins such as sarcomeric α-actinin and that they have the morphological appearance of myocytes including well-defined sarcomeric structures, as we have also confirmed in this study. Further, the unique distribution of PdCMs and feasibility of light-pacing these cells in LA and LV may provide an opportunity to develop a cell-type specific biological pacemaker to correct sinus node dysfunction.

From our studies, we can also conclude that the majority of PdCMs lose their ability to synthesise adrenaline at some time during development. However, it is possible that low-level expression of the synthesising enzyme may exist but is not be detected by the staining methods. Interestingly, Pnmt staining was observed in some PdCMs (~10% of isolated ventricular PdCMs) (Fig. 2e). A similar observation is also described in our previous paper in which immunofluorescent co-staining with Pnmt and sarcomeric α-actinin showed apparent Pnmt expression within some cardiomyocytes^5^. The functional relevance of the Pnmt-positive PdCMs cells in the adult heart is unclear. These cells may play a role in localized adrenaline production if they have endocrine capability, or they may have an unrelated role. Further investigation and clarification are needed to determine between these possibilities.

Nevertheless, it is clear that a substantial number of ICA cells is present in adult heart^7^. Recently, we suggested that Pnmt^+^ primer cells may have therapeutic potential, since these progenitor cells can be a new source for neuro/myocardial regeneration^1^. The regenerative capability of the heart is highly clinically relevant, as is the inability of adult heart to replace the lost cardiomyocytes after cardiac injury contributing to the ongoing encumbrance of heart failure. Recent studies have indicated the potential importance of these cells for cardiac regeneration and sympathetic re-innervation following cardiac transplantation^6^,^7^,^8^. The restoration of cardiac sympathetic activity in transplanted heart is tightly coupled with an increase in the number of neural crest-derived adrenergic cells^8^.

These studies have provided important new information to the active field of cardiac regeneration by identifying a role of sympathetic nerves in mammalian heart regeneration. However, the molecular mechanisms of the sympathetic nerve–mediated regulation of cardiac regeneration were not defined. Further work will be required to determine whether and how sympathetic innervation might influence cardiac regeneration in the adult heart. Consequently, it is important to develop a better understanding of the physiological roles of ICA cells and PdCMs *in vivo*^8^. Thus the current study may lead to another important route for cardiac regenerative medicine. The specific type of stem cells needed for successful cardiac repair/regeneration strategies has not yet been established, although several different cell types are currently under exploration for this purpose. PdCMs identified in this study may lead to another important avenue for cardiac regenerative medicine based on the possible adrenergic lineage. Our new mouse model provides a unique model system for such studies.

### Limitations and future perspectives of the study

The physiological roles of the PdCMs and their implications in diseases and therapeutics require further investigation. Cre-mediated recombination occurs early in development, which requires expression from an activated Pnmt gene, but Pnmt expression does not need to remain active in the cells. Indeed there is evidence from this and prior studies that many PdCMs do not appear to express appreciable amounts of Pnmt by adult stages of development, indicating that Pnmt may be a transient marker for this subpopulation (PdCMs). Future studies using inducible Cre/loxP systems such as the CreERT2/loxP system, enables tamoxifen-inducible temporal control of gene deletion/expression in adulth mice.

The optogenetic manipulations in cardiac tissue will provide great opportunities for examining many hypotheses that were previously untestable as a result of technical limitations associated with traditional electrophysiological techniques. To date most of the optogenetic studies have used optogenetic stimulation, but optogenetic inhibition is likely to be a powerful apprach to determine the behaviors of specific heart cells as optogenetic stimulation. In addition, combining optogenetics with *in vivo* monitoring techniques such as *in vivo* electrophysiology and imaging, allows for demonstration of cell function *in vivo*. The ever-increasing methods for targeted genetic manipulations of heart cells as well as the continued development and refinement of optogenetic methods will be unprecedented.

## Materials and Methods

### Experimental Procedures

The mouse line was generated by crossing Pnmt-Cre mice^4^ with B6.Cg-*Gt (ROSA)26Sor*^*tm27.1(CAG-COP4H134R/tdTomato) Hze*^/J strain (Stock No. 012567, Jackson Labs)^19^.

The protocol for generating and maintaining the mice were approved by the Oxford University Ethical Review Committee (Pharmacology subcommittee) and Home Office (Project licence: 30/3340). We confirm that all methods were performed in accordance with the relevant UK Home Office and institutional guidelines and regulations.

#### Immunohistology

All mice were sacrificed via cervical dislocation in accordance to the Animals Scientific Procedures Act (1986). The heart was isolated and cannulated at the aorta to allow retrograde perfusion of solution. Phosphate-buffered saline (PBS) was flushed through the heart to remove any residual blood, and the SA and AV node regions were isolated if required. Adrenal glands were also isolated to serve as positive controls. Isolated tissues required for immunohistochemistry were embedded in Optimal Cutting Temperature (OCT) compound and frozen with isopentane in dry ice for 30 minutes. 10 μm coronal or transverse sections were then taken using a Leica CM 3050 S cryostat, which were then mounted on Polysine microscope slides.

After mounting of specimens, all slides were washed in PBS and fixed with 4% paraformaldehyde (PFA) (Sigma-Aldrich). Slides were again washed with PBS, and the cell membranes permeabilised with Triton-X100 (ThermoFisher). Specimens were blocked with goat serum, and incubated with primary antibodies overnight. The following day, slides were washed with PBS and incubated with secondary antibodies for 2 hours. Slides were washed with PBS, mounted in Vectashield mounting medium (Vector Labs) and sealed with a coverslip and nail varnish. Fluorescence images were obtained using Olympus FV1000 Confocal and Zippy Moving-stage Fluorescent microscopes. Primary antibodies: Anti-Pnmt (monoclonal rabbit, Abcam, UK), 1:50; Anti-HCN4 (polyclonal rabbit, Alamone, Israel), 1:20; Anti-ChR2 (monoclonal mouse, Progen, UK), 1:500; Anti-α-actinin (monoclonal mouse, Sigma Aldrich, UK), 1:400. Goat secondary antibodies (Invitrogen) were used as 1:500 or 1:1000 dilutions.

#### Cell isolation

Single ventricular myocytes were isolated from the hearts by collagenase digestion in a similar manner as previously described^20^. In brief, the mouse was sacrificed by Schedule 1 killing and the heart was immediately removed and cannulated onto the Langendorff system. The heart was perfused with isolation solution at a rate of 3.5~4 ml/min (37 °C). Brief perfusions of the heart with isolation solution flushed any blood inside the heart, prior to perfusions with collagenase solution (collagenase 0.8 mg/ml, CLS-2 (Worthington Ltd, UK) for 5–7 mins. The ventricles were cut and chopped into small pieces in normal Tyrode solution rich in taurine, suspended gently and cells collected by filtration.

#### 3D Image Reconstruction

A series of microscopic images were fused to constitute a single slice of the mouse heart using Microsoft Image Composite Editor, an advanced panoramic image stitcher. For each slice, the positively stained tissue was determined using a semi-automatic intensity-based method: the sites with clusters of the positively stained tissue were manually labelled; the positively stained sites were defined as the pixels with intensities exceeding a predefined threshold. Rigid registration (stiff rotation and translation) was applied to properly align the fused heart slice images based on landmarks using the TrakEM2^22^, an open source plug-in in Fiji^23^. Each slice was smoothed with a Gaussian filter and subsequently down sampled to minimize image noise. The registered slices were then volume rendered by MATLAB (MATLAB^®^. *Version 8.3.0.532)*. The raw image volume was 736 × 901 × 23 pixels. Since the image slices were not evenly spaced, intensity-based linear interpolation along the image alignment direction was carried out to account for the missing gaps using MATLAB. The final reconstructed 3D volume was 736 × 901 × 51 pixels, and visualised in Paraview^24^, an open source application for data analysis and visualisation.

#### Optical stimulation of ChR2 light-sensitive channel

Whole hearts, tissues or single cells were paced through the activation of ChR2 light-sensitive channels. This was achieved by the delivery of 470 nm blue light pulses (2–10 ms pulse width) generated by OptoFlash (Cairn Research). Pulses were triggered by a 1401 digitiser and Spike 2 software (Cambridge Electronic Design). Approximate blue light intensity was measured with a 818-ST2 Wand Detector connected to a 843 R Power meter (both Newport Corporation, CA, USA) and expressed normalised for the area being illuminated through simulating the average light intensity reached to the surface of the tissue by mimicking the distance of fiber-heart.

#### Electrocardiography (ECG)

To monitor cardiac rhythms we carried out *ex vivo* ECG analysis on langendorff perfused hearts. RR interval, P wave duration, PR interval, QRS and QT durations were measured.

#### Monophasic action potential (MAP) recording with programmed light stimulation (PLS) and programmed electrical stimulation (PES)

Langendorff-perfused *ex vivo* hearts from *Pnmt*^*Cre/ChR2*^ mice were subjected to PLS or PES while MAPs were being measured from an electrode placed on the left ventricle of the heart. Three protocols were carried out as follows: (a) continuous pacing protocol: Stimuli were delivered continuously with a constant frequency. (b) S1S2 pacing protocol: a pacing train of eight stimuli (S1) was delivered at a basic cycle length of 100 ms, with a single (S2) premature extra stimulus introduced at progressively shorter intervals until arrhythmia was induced or the ventricular refractory period was reached. (c) burst pacing protocol: 50 stimuli with progressively reduced stimulus interval (from 100 ms to 30 ms) were delivered. Ventricular tachycardia was defined as six or more consecutive premature ventricular waveforms; tachycardia with regular waveforms defined as VT and VF was characterized by irregular fibrillating waveforms.

#### Optical voltage and Ca^2+^ imaging

To assess global Ca^2+^ and voltage characteristics of atrial tissue or ventricle, either Langendorff perfused heart or atria were prepared. Blebbistatin, a myosin II inhibitor, was used to stop contractions and avoid movement artefacts. Tissue was incubated with blebbistatin at a concentration of 10 μM for 45 min. After cessation of contractions, tissue was loaded with the acetoxymethyl (AM) ester of rhod-2 (45 min loading of 4 μM dye in static, oxygenated solution) to measure cellular Ca^2+^, and with RH237 (applied to tissue by bolus of 36 μl at 2 mM concentration over 5–7 mins) to measure membrane voltage. Ca^2+^ and voltage dyes were both excited by light at 530 nm, and emitted light at 581 nm and 782 nm respectively. Signals were filtered to separate Ca^2+^ and voltage channels with a 570 nm long pass optical filter and a 625 nm long pass dichroic mirror and, followed by analysis using Metamorph software (Molecular Devices). Light pacing pulses were delivered to the epicardial surface of the atrium and pacing protocols were applied. Tissues were imaged at a frame rate of 1 kHz for 2–3 s during and immediately after each pacing interval is applied. A temperature of 32 °C was maintained throughout this method. Image files were analysed to assess wave-speed, changes in action potential and calcium transient characteristics.

#### Contraction measurement

Mechanical properties of ventricular myocytes were assessed using an IonOptix MyoCam system (IonOptix Corp., Milton, MA, USA). Cells were placed in a chamber mounted on the stage of an inverted microscope (Model: Olympus, IX-70, Leeds Precision Ltd, Minneapolis, MN, USA) and superfused (25 C) with a buffer containing (in mM): 131 NaCl, 4 KCl, 1 CaCl2, 1MgCl2, 10 glucose, 10 HEPES, at pH 7·4. The cells were field or light stimulated with suprathreshold voltage at a frequency of 0·5–1 Hz and 3-ms duration. For the measurements of cell contraction, the sarcomeres of the cells were visualised via a 40x oil objective lens using IonOptix MyoCam (IonOpix Corp.), which sampled images at 240 Hz frequency. The optical density traces raised from the alternating light and dark bands of the contractile machinery and showed sinusoidal pattern with wavelength of the sine representing sarcomere length, the traces were then computed into a signal of sarcomere length by application of a fast Fourier transform (FFT) by the IonWizard sarcomere length acquisition software (IonOptix Corp.). Vertical cursors defined the length of myocytes used for this calculation, and always included a minimum of 7 sarcomeres. Length measurements were calibrated using a stage micrometre with 2 μm graduations such that the number of pixels/μm recorded by the MyoCam could be entered into the software as a fixed values. Amplitude of sarcomere shortening was calculated by deduction of systolic from diastolic sarcomere length and expressed either in microns or as a percentage of resting sarcomere length. Analysis was performed using IonWizard 5 software (IonOptix Corp.). All values represented an average of 10 contractions.

### Data Analysis and Statistical Methods

In the analysis of the continuous experimental data, values are reported as mean ± SEM of n experiments, and the observations were thought to be close to a normal distribution sufficiently to permit use of parametric tests. Paired or unpaired *t* tests were used as appropriate. All *p* values are 2 sided, the statistical software used to generate the results is SigmaStat software.

## Additional Information

**How to cite this article**: Wang, Y. *et al*. Optogenetic Control of Heart Rhythm by Selective Stimulation of Cardiomyocytes Derived from Pnmt^+^ Cells in Murine Heart. *Sci. Rep.* **7**, 40687; doi: 10.1038/srep40687 (2017).

**Publisher's note:** Springer Nature remains neutral with regard to jurisdictional claims in published maps and institutional affiliations.

## Supplementary Material

Supplementary Information

Supplementary Video 1

Supplementary Video 2

Supplementary Video 3
