# Supplementary material for: Optogenetic Control of Heart Rhythm by Selective Stimulation of Cardiomyocytes Derived from Pnmt+ Cells in Murine Heart
Source: Sci Rep. 2017 Jan 13;7:40687. doi: 10.1038/srep40687 (PMC5234027; doi:10.1038/srep40687)
Supplement: Supplementary Information [file srep40687-s1.pdf]

Title page

Optogenetic Control of Heart Rhythm by Selective Stimulation of Cardiomyocytes Derived from Pnmt^{+} Cells in Murine Heart

Yanwen Wang[1*], Wee Khang Lin[1*], William Crawford[2], Haibo Ni[3], Emma L Bolton[1], Huma Khan[1], Julia Shanks[4], Gil Bub[4], Xin Wang[6], David J Paterson[4], Henggui Zhang[3], Antony Galione[1], Steven N Ebert[†5],Derek A Terrar[†1] and Ming Lei[†1]

1. Department of Pharmacology, University of Oxford, Oxford, UK
2. University of Queensland, Brisbane, Australia
3. School of Physics and Astronomy, University of Manchester, Manchester, UK
4. Department of Physiology, Anatomy and Genetics, University of Oxford, Oxford, UK
5. Burnett School of Biomedical Sciences, College of Medicine, University of Central Florida, Orlando, USA
6. Faculty of Life Science, University of Manchester, Manchester, UK

*Contributed equally to this work

[†] Senior Authors

Correspondence to: Dr. Ming Lei: Department of Pharmacology, University of Oxford, Mansfield Road, OX1 3QT.

Fax: 00441865271850; Tel: 00441865 271850; E-mail: ming.lei@pharm.ox.ac.uk

Supplementary Information

Legends for online videos

Online Video 1. 3D reconstruction of the distribution of ChR2/tdTomato positive cells in the ventricle using customized computer algorithms to generate three-dimensional representations of the ChR2/tdTomato staining patterns.

Online Video 2. Langendorff-perfused *ex vivo* $Pnmt^{Cre/ChR2}$ heart subjected to programmed light stimulation. Light pulses delivered to the LA or LV of a $Pnmt^{Cre/ChR2}$ heart evoked ECG spikes indicating that light pulses were able to induce a new heart rhythm by overriding intrinsic sinus rhythm, but light pulses on RV could not override the sinus rhythm

Online Video 3. Contractions triggered by light pulses applied to single NEPC-derived ventricular cardiomyocytes isolated from a $Pnmt^{Cre/ChR2}$ heart.
